# Bacteriological Safety of Blood Collected for Transfusion at University of Gondar Hospital Blood Bank, Northwest Ethiopia

**DOI:** 10.1155/2013/308204

**Published:** 2013-06-20

**Authors:** Hailegebriel Wondimu, Zelalem Addis, Feleke Moges, Yitayal Shiferaw

**Affiliations:** ^1^Debre Tabor Health Science College, P.O. Box 83, Debre Tabor, Ethiopia; ^2^School of Biomedical and Laboratory Sciences, College of Medicine and Health Sciences, University of Gondar, P.O. Box 196, Gondar, Ethiopia

## Abstract

*Background*. Transfusion associated bacterial infection has remained more frequent with a sever risk of morbidity and mortality. This study assessed the bacteriological safety of blood collected for transfusion. *Method*. A cross-sectional study was conducted at University of Gondar hospital blood bank from December 2011 to June 2012. Bacterial isolation, identification, and antimicrobial susceptibility tests were done as per the standard procedure. Chi-square test and *P* value were used to assess associations between risk factors and the bacterial isolation rate. *Results*. Twenty-one (15.33%) blood units were found contaminated with bacteria, and 95.24% contamination was due to external sources. The commonly isolated bacteria were *Staphylococcus aureus*, Coagulase negative *Staphylococci*, *Escherichia coli*, *Klebsiella* species, *Streptococci* species, *Enterobacter* species, and *Citrobacter* species. All of the bacteria isolated were 100% sensitive to Gentamicin, Chloramphenicol, Amoxicillin, and Doxycycline. Multiple antimicrobial resistances were observed in 66.7% of the isolates. Not using glove by phlebotomist, touching disinfected phlebotomy site and double puncture at the same hand or both hands of a donor were found to be risk factors for bacterial contamination. *Conclusion*. Bacterial contamination of blood to be transfused is a common problem in the hospital. So attention should be given to activities performed at the blood bank for safe transfusion practices.

## 1. Introduction

The development of modern blood transfusion medicine represents one of the greatest achievements of medicine in the 20th century with about 75 million blood units being collected and transfused yearly [1, 2]. Although millions of lives are saved by blood transfusion, limited access to transfusion and the provision of unsafe blood is putting millions of people at risk of transfusion transmissible infections (TTI) [1, 3, 4].

Due to stringent donor selection, improved mandatory tests, and close surveillance of new emerging infections, the risk of TTI in developed countries is very low [5], but blood safety remains an important public health concern in Africa where lack of availability and provision of unsafe blood adversely impacts morbidity and mortality in the region [6, 7].

Transfusion associated bacterial infection (TABI) has remained more frequent than viral infections and is associated with high mortality due to rapid occurrence of septic shock [8–11]. Contaminated blood units may contain a numbers of virulent bacteria as well as endotoxins that are considered to be fatal to the recipient [12, 13]. The potential sources of bacterial contamination of blood collected for transfusion are skin flora introduced at the time of phlebotomy and from bacteria in the donor's blood because of an underlying condition causing donor bacteremia [9, 14, 15].

In many African countries concern over TTIs usually focuses on viral risk, yet the risk of bacterial contamination, incurred during collection and processing, is 2500 times higher than in developed countries [4, 16]. Moreover countries in the region relay only on visual examination of the blood bag for evidence of hemolysis just prior to transfusion with its defect as a screening method to check for bacterial contamination [10, 17]. This makes the burden of TABI be more prevalent. 

Studies from Africa including from Kenya [16], Nigeria [18], Ghana [19], and elsewhere in the world [13, 20–23] have indicated the magnitude of the problem, but, bacteriological safety of blood for transfusion is not well addressed in Ethiopia in general and in the study area in particular. So the aim of this study was to assess rate of bacterial contamination of blood collected for transfusion and to determine the antimicrobial susceptibility pattern of the isolated bacteria.

## 2. Materials and Methods

### 2.1. Study Area and Study Design

A cross-sectional study was conducted from December 2011 to June 2012 at University of Gondar hospital blood bank which is a teaching hospital that provides health service to over five million inhabitants in Northwest Ethiopia. According to the data obtained from the blood bank the average annual number of blood units collected is 2000 and majority of which are provided for surgical and gynecological cases.

### 2.2. Sample Size Determination and Sampling Procedure

The sample size was calculated based on assumption of prevalence of bacteria isolated from blood for transfusion in Kenya and Nigeria with each reporting 8.8% [16, 18]. With 5% margin of error and 95% confidence interval (alpha = 0.05), the actual sample size for the study was computed using one sample population proportion formula (Cochran's sample size formula) as indicated in the following:

(1) $$\begin{array}{c} n=\frac{{\left(Za/2\right)}^{2}pq}{W^{2}}. \end{array}$$

Using systematic random sampling, a total of 137 blood donors and blood units were investigated. 

### 2.3. Data Collection and Laboratory Procedure

#### 2.3.1. Sociodemographic Characteristics and Other Factors

The sociodemographic characteristics of blood donors (age, sex, occupation, and others) and other factors like type of blood donors, use of glove by phlebotomist during blood collection, and double puncture at the same hand or both hands of a donor were collected using interview administered structured questionnaire and direct observation of bleeding procedures. 

#### 2.3.2. Sample Collection

Five milliliter of venous blood was collected directly from the donors and another 5 mL of blood was taken from the blood units ready for transfusion. Blood units collected from the sampled donor were mixed and kept inverted down for 30 to 45 minutes. This allows some sediments of blood (to maximize sensitivity of bacterial isolation) to slip out of the blood bag into the part of the septum at which the sample will be collected. After the period of sedimentation the septum near to the bag was clipped in order to block blood leakage through the septum that will be punctured. Finally 5 mL of blood was drawn from the part of septum already prepared for this purpose by sterile syringe with needle after disinfection with tincture of iodine [24]. 

#### 2.3.3. Bacterial Isolation and Identification

Blood samples were inoculated in duplicate on to 45 mL of brain heart infusion (BHI) medium, incubated at 37°C, and observed daily after 48 hours of incubation for 5 to 7 consecutive days for presence of turbidity, hemolysis, and color changes which are evidence of microbial growth. Whenever visible sign of growth appears, small amount of the cultures was subcultured on to blood agar plate (BAP) and MacConkey (MAC) agar and incubated for 24 to 48 hours at 37°C. Pure colonies were examined by Gram's staining and further identification was made using different biochemical tests including catalase and coagulase tests for Gram-positive bacteria and hydrogen sulphide production (H_2_S), indole test, citrate utilization, lysine decarboxylase (LDC) test, gas production, and carbohydrate metabolism for Gram-negative bacteria [24–26]. 

#### 2.3.4. Antimicrobial Susceptibility Test

Isolated bacteria were tested for their susceptibility pattern according to Kibry-Bauer disk diffusion method on Muller-Hinton agar [26] using a panel of 14 antimicrobials including Ampicillin (10 *μ*g), Gentamicin (10 *μ*g), Tetracycline (30 *μ*g), Ciprofloxacin (5 *μ*g), Chloramphenicol (30 *μ*g), Trimethoprim-sulfamethoxazole (25 *μ*g), Ceftriaxone (30 *μ*g), Amoxicillin (30 *μ*g), Penicillin G (10 units), Methicillin (5 *μ*g), Vancomycin (30 *μ*g), Clindamycin (2 *μ*g), Erythromycin (15 *μ*g), and Doxycycline (30 *μ*g). Pure colonies of the test organism were taken using a sterile wire loop and emulsified in 3-4 mL of sterile nutrient broth. Bacterial suspensions were compared with 0.5 McFarland standard. Then a sterile cotton swab was dipped into the suspension and bacteria were inoculated onto the Muller-Hinton agar. The discs were placed on to the surface of inoculated media by using disc dispenser and incubated for 24 hr at 37°C. Results were read and recorded by measuring inhibition zone diameters to the nearest millimeter and interpreted after comparing with the standards, and isolates were classified as susceptible, intermediate, or resistant to the tested antibiotics [26].

### 2.4. Data Quality Control

All culture media were prepared following the manufacturer's instructions. Each batch of the prepared media was checked for sterility by incubating a sample medium at 37°C for 24 hr. Known bacterial species were inoculated and incubated at 37°C for 24 hr for the performance check [26]. *E. coli* ATCC25922 and *S. aureus *ATCC25923 sensitive to all antimicrobial agents were used as control strains. 

### 2.5. Data Analysis

Statistical analysis was done using SPSS version 16.00 statistical software. Frequency and percentage were employed to summarize the results and presented in tables and graphs. Chi-square (*χ* ^2^) and *P* value were used to determine the association and strength of risk factors with the bacterial isolation rate from the collected blood for transfusion. A *P* value of less than 0.05 was considered as statistically significant.

### 2.6. Ethical Consideration

Ethical approval of the research was obtained from Ethical Review Committee of School of Biomedical and Laboratory Sciences College of Medicine and Health Sciences, and official letter was directed to University of Gondar hospital blood bank. Informed consent was obtained from the blood donors. Donor with bacteremia was contacted by the address registered on the donor's card and advised to communicate with clinicians.

## 3. Results

### 3.1. Sociodemographic and Related Characteristics of Blood Donors

A total of 137 blood donors and blood units collected from them were included in this study. The mean age of the study participants was 30.3 years with a standard deviation of 10.1, and the majority, 59.1%, of them were under the age group of 22−35 years. Males comprise about 81% of the study participants. Large number of the donors, 72.3%, 51.1%, 75.2%, and 44.5%, were urban residents, married, Christians by religion, at level of college/university in education, respectively. About 77% of the donors were family replacement donors, and 72.3% of the donors have no history of previous donation (Table 1).

### 3.2. Rate of Bacterial Isolation

**Figure 1:**
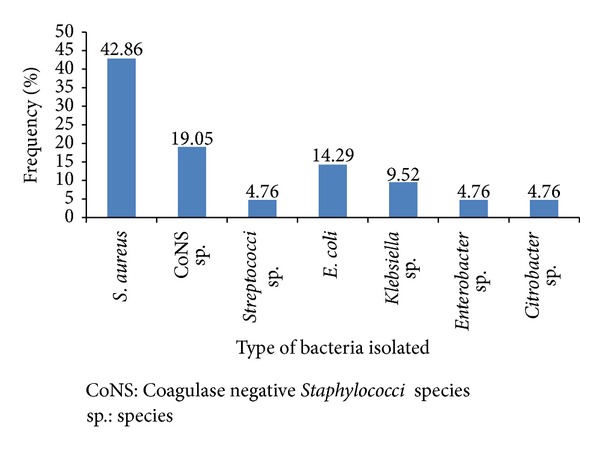
Type and frequency of bacteria isolated from blood units collected for transfusion at University of Gondar hospital blood bank, Northwest Ethiopia, 2012.

Over the study period, 21 blood units, out of 137, were found to be contaminated with bacteria making the bacterial isolation rate of 15.3%. Only in a single instant bacteria were isolated from the donor as well as from the respective blood unit, representing 1/21 (4.8%) of contaminated blood units. The majority, 66.7% (14/21), of the organisms isolated were Gram-positive mainly *S. aureus *(42.9%), Coagulase negative *Staphylococci* species (19.05%), and *Streptococci *species (4.8%). Gram-negative bacteria isolated include *E. coli *(14.2%), *Klebsiella *species (9.52%), *Enterobacter* species (4.8%), and *Citrobacter* species (4.8%) (Figure 1).

### 3.3. Phlebotomy Procedures and Association with Rate of Bacterial Contamination

During collection of blood for transfusion only 70% alcohol was used as a disinfectant, and about 70.8% (97/137) of sampled blood units were collected without using glove by the phlebotomist. Moreover 10.2% (14/137) of sampled blood units were collected from the donors whose disinfected phlebotomy site had been touched by the hand of blood collectors and 7.3% (10/137) had been collected by double puncture at the same hand or both hands of a donor. When these activities were tested for association with rate of bacterial contamination not using glove by phlebotomist during blood collection, touching disinfected phlebotomy site and double puncture at the same hand or both hands of a donor have shown statistically significant association with bacterial contamination (Table 2).

### 3.4. Antimicrobial Susceptibility Test Result

The bacterial isolates showed diverse susceptibility patterns to the antibiotics tested. All the bacteria isolated were 100% sensitive to Gentamicin, Chloramphenicol, Amoxicillin, and Doxycycline. All Gram-positive isolates were 100% sensitive to Vancomycin, Ciprofloxacin, and all Gram-negative isolates were 100% sensitive to Ceftriaxone and Erythromycin. However, all Gram-negative bacteria were 100% resistant to Penicillin G (Table 3). Multiple antimicrobial resistances were observed in 66.7% (14/21) of the isolated bacteria.

## 4. Discussion

The present study showed 15.33% bacterial contamination rate of whole blood collected for transfusion at University of Gondar hospital blood bank which is strikingly higher than the rates detected in United kingdom (0.19%), Canada (0.2 to 0.4%), and Japan (6.3%) [12, 21, 27]. The lower prevalence in other countries may be due to close surveillance of emerging infections and the meticulous care of blood collection procedure with stringent donor selection and presence of efficient infection prevention controls protocol, which are too poor in developing countries [1, 28, 29]. The current result is also higher than rates reported from African countries including Ghana (9%), Nigeria (8.8%), and Kenya (8.8%) [16, 18, 19]. The higher prevalence in our study may be due to the unusual practice of glove use and touching the site of phlebotomy after disinfection as well as double puncture which are observed in this study area even though such factors are not well reported from other related studies, but this rate is relatively lower than reports from Ghana where contamination rate was 17.5% [30]. 

The most frequently isolated bacteria were *S. aureus* followed by Coagulase negative *Staphylococci, E. coli* and *Klebsiella* species. Similar findings were reported elsewhere [14, 16, 18, 22, 27, 30, 31].

The potential source of bacterial contamination of blood collected for transfusion is either bacteria in the donor's blood because of an underlying condition causing donor bacteremia or external contaminants introduced at the time of phlebotomy [9, 14, 15]. The source of bacterial contaminant of sampled blood units accounted by the donor bacteremia was only 4.76%. However, 95.21% of contaminations were contributed by contaminants introduced at the time of phlebotomy. Different reports indicated that proper blood donor skin disinfection has long been recognized as a definite way to reduce blood contamination [27, 32]. This is strongly supported by this study as glove use, touching the disinfected phlebotomy site, and double puncture have shown statistically significant association with the bacteriological culture positivity of blood units. In addition the isolates obtained in our study were mostly skin associated organisms, and total *coli* form groups, which are often considered contaminants related to procedure during blood collection rather than donor bacteremia. 

The antimicrobial resistance pattern observed in this study was in agreement with reports from Ghana, where most of the isolated organisms showed to be susceptible to Gentamicin and Erythromycin while they were resistant to Penicillin G and Tetracycline. The rate of multiple antimicrobial resistances observed was also in agreement with these studies [19, 30].

The resistance rates in the organisms isolated highlight the growing problem of antimicrobial resistance. The risks of transfusing contaminated donor blood are high, and transfusing blood with drug resistant strains of bacteria may worsen the difficulty of the already sick and the immunocompromised individuals as these organisms are capable of causing serious risk of fatality when transfused to patients [32].

## 5. Conclusion 

Knowledge of the prevalence of bacterial contamination of blood for transfusion and the sources or causes of contamination in different parts of the world, particularly in Africa, is important for the planning of preventive measures at blood transfusion centers and the reduction of TABI. 

From this study it can be concluded that bacterial contamination of donated blood is highly prevalent in the study area which indicates a potential risk of health care associated infection to patients. This has been approved by study conducted in USA [33]. Moreover high resistance patterns observed for single and multiple antimicrobials are also a great concern that needs urgent attention. Therefore, this study calls a need for supervision and corrective actions for mistakes that are made during blood collection, for introduction of policies for safe transfusion practices, and further research to clarify the extent and nature of the problem.

## Figures and Tables

**Table 1:** Sociodemographic variables of blood donors at University of Gondar hospital blood bank, Northwest Ethiopia, 2012.

Variables	Frequency (*n*)	Percent (%)
Age		
Under 21	19	13.9
22–35	81	59.1
36–49	27	19.7
Above 50	10	7.3
Sex		
Male	111	81.0
Female	26	19.0
Place of residence		
Rural	38	27.7
Urban	99	72.3
Secondary school	26	19.0
College/university	61	44.5
Type of donor		
Volunteer	27	19.7
Replacement	106	77.4
Paid	4	2.9
Number of previous donation		
0 times	99	72.3
Once	16	11.7
Twice	3	2.2
Three times	2	1.5
More than three times	17	12.4

**Table 2:** Procedural activities performed during the collection for transfusion and their association with rate of bacterial contamination.

Activities	Culture results		*χ* ^2^ (*P* value)
	Positive	Negative	
Glove use			
Yes	2	38	4.64 (0.031)
No	19	78	
Touching disinfected phlebotomy site			
Yes	11	3	48.05 (0.000)
No	10	113	
Double puncture			
Yes	9	1	46.34 (0.000)
No	12	115	
Use of tincture of iodine for disinfection			
Yes			
No	21	116	

**Table 3:** Antimicrobial susceptibility pattern bacteria isolated from blood units collected for transfusion.

Drugs tested	Species isolated *n* (%)						
	*S. aureus *	CoNS	*Streptococci *	*E. coli *	*Klebsiella *	*Enterobacter *	*Citrobacter *
AMP							
S	5 (55.6)	2 (50)	1 (100)	2 (66.7)	2 (100)	0 (0)	1 (100)
I	0 (0)	0 (0)	0 (0)	0 (0)	0 (0)	1 (100)	0 (0)
R	4 (44.4)	2 (50)	0 (0)	1 (33.3)	0 (0)	0 (0)	0 (0)
CN							
S	9 (100)	4 (100)	1 (100)	3 (100)	2 (100)	1 (100)	1 (100)
I	0 (0)	0 (0)	0 (0)	0 (0)	0 (0)	0 (0)	0 (0)
R	0 (0)	0 (0)	0 (0)	0 (0)	0 (0)	0 (0)	0 (0)
MET							
S	8 (88.9)	4 (100)	1 (100)	NA	NA	NA	NA
I	1 (11.1)	0 (0)	0 (0)	NA	NA	NA	NA
R	0 (0)	0 (0)	0 (0)	NA	NA	NA	NA
TE							
S	7 (77.8)	4 (100)	1 (100)	2 (66.7)	1 (50)	1 (100)	0 (0)
I	0 (0)	0 (0)	0 (0)	1 (33.3)	0 (0)	0 (0)	1 (100)
R	2 (22.2)	0 (0)	0 (0)	0 (0)	1 (50)	0 (0)	0 (0)
VA							
S	9 (100)	4 (100)	1 (100)	NA	NA	NA	NA
I	0 (0)	0 (0)	0 (0)	NA	NA	NA	NA
R	0 (0)	0 (0)	0 (0)	NA	NA	NA	NA
P							
S	4 (44.4)	2 (50)	0 (0)	0 (0)	0 (0)	0 (0)	0 (0)
I	0 (0)	0 (0)	0 (0)	0 (0)	0 (0)	0 (0)	0 (0)
R	5 (55.6)	2 (50)	1 (100)	3 (100)	2 (100)	1 (100)	1 (100)
CIP							
S	9 (100)	4 (100)	1 (100)	0 (0)	1 (50)	0 (0)	0 (0)
I	0 (0)	0 (0)	0 (0)	2 (66.7)	0 (0)	0 (0)	0 (0)
R	0 (0)	0 (0)	0 (0)	1 (33.3)	1 (50)	1 (100)	1 (100)
C							
S	9 (100)	4 (100)	1 (100)	3 (100)	2 (100)	1 (100)	1 (100)
I	0 (0)	0 (0)	0 (0)	0 (0)	0 (0)	0 (0)	0 (0)
R	0 (0)	0 (0)	0 (0)	0 (0)	0 (0)	0 (0)	0 (0)
SXT							
S	8 (88.9)	4 (100)	1 (100)	3 (100)	1 (50)	0 (0)	1 (100)
I	1 (11.1)	0 (0)	0 (0)	0 (0)	1 (50)	1 (100)	0 (0)
R	0 (0)	0 (0)	0 (0)	0 (0)	0 (0)	0 (0)	0 (0)
DA							
S	7 (77.8)	4 (100)	0 (0)	0 (0)	1 (0)	0 (0)	0 (0)
I	0 (0)	0 (0)	1 (100)	0 (0)	0 (0)	0 (0)	1 (100)
R	2 (22.2)	0 (0)	0 (0)	3 (100)	1 (50)	1 (100)	0 (0)
CRO							
S	9 (100)	2 (50)	1 (100)	3 (100)	2 (100)	1 (100)	0 (0)
I	0 (0)	2 (50)	0 (0)	0 (0)	0 (0)	0 (0)	1 (100)
R	0 (0)	0 (0)	0 (0)	0 (0)	0 (0)	0 (0)	0 (0)
AMC							
S	9 (100)	4 (100)	1 (100)	3 (100)	2 (100)	1 (100)	1 (100)
I	0 (0)	0 (0)	0 (0)	0 (0)	0 (0)	0 (0)	0 (0)
R	0 (0)	0 (0)	0 (0)	0 (0)	0 (0)	0 (0)	0 (0)
E							
S	7 (77.8)	4 (100)	1 (100)	3 (100)	2 (100)	1 (100)	1 (100)
I	1 (11.1)	0 (0)	0 (0)	0 (0)	0 (0)	0 (0)	0 (0)
R	1 (11.1)	0 (0)	0 (0)	0 (0)	0 (0)	0 (0)	0 (0)
DO							
S	9 (100)	4 (100)	1 (100)	3 (100)	2 (100)	1 (100)	1 (100)
I	0 (0)	0 (0)	0 (0)	0 (0)	0 (0)	0 (0)	0 (0)
R	0 (0)	0 (0)	0 (0)	0 (0)	0 (0)	0 (0)	0 (0)

AMP: Ampicillin; CN: Gentamicin; TE: Tetracycline; CIP: Ciprofloxacin; C: Chloramphenicol; SXT: Trimethoprim-sulfamethoxazole; CRO: Ceftriaxone; AMC: Amoxicillin; P: Penicillin G; MET: Methicillin; VA: Vancomycin; DA: Clindamycin; E: Erythromycin; DO: Doxycycline; NA: not applicable; CoNS: Coagulase negative *Staphylococci* species; S: sensitive; I: intermediate; R: resistance.
